# Evaluation of solar photovoltaic carport canopy with electric vehicle charging potential

**DOI:** 10.1038/s41598-023-29223-6

**Published:** 2023-02-06

**Authors:** Hoda Fakour, Moslem Imani, Shang-Lien Lo, Mei-Hua Yuan, Chih-Kuei Chen, Shariat Mobasser, Isara Muangthai

**Affiliations:** 1 International Program for Sustainable Development, International College of Practice and Education for the Environment, Chang Jung Christian University, No.1, Changda Rd., Gueiren District, Tainan City, 71101 Taiwan; 2 Graduate Institute of Environmental Engineering, National Taiwan University, No. 1, Sec. 4, Roosevelt Rd, Taipei, 106 Taiwan; 3 Water Innovation, Low-Carbon and Environmental Sustainability Research Center, College of Engineering, National Taiwan University, Taipei, 106 Taiwan; 4 Research Center for Environmental Changes, Academia Sinica (AS), Academia Rd, No. 128, Sec. 2, Taipei, 115 Taiwan; 5 Department of Environmental Engineering, National Ilan University, Yilan City, 26047 Taiwan; 6 College of Engineering, Center for the Environment and Laboratory of Renewable Resources Engineering, Purdue University, 610 Purdue Mall, West Lafayette, IN 47907 USA; 7 Warash School of Maritime Science and Engineering, Solent University, Southampton, UK

**Keywords:** Energy science and technology, Climate-change mitigation

## Abstract

While sustainable mobility and decarbonization of transportation sector are among the most comprehensive solutions to the problem of climate change, electric vehicles (EV) are becoming increasingly popular as the future mode of transport. In this study, the integration of a solar carport canopy to a potential EV charging station is analyzed using various operating conditions. A detailed analysis has been provided for the carport located in southern Taiwan, Kaohsiung city, where electricity generation, emission impacts, and financial analysis of the solar EV charging station are discussed. The results of a case study showed a potential of 140 MWh/year of solar energy yield, which could provide solar electricity of more than 3000 vehicles per month with 1-h parking time, generating 94% lower total carbon dioxide emission than the electricity produced from traditional grid methods. Taken into account the impact of carbon tax implementation on driver economics, the results demonstrated the viability of such photovoltaic (PV)-based charging stations, particularly for possible higher carbon tax scenarios in the future. The presented results can be implemented on a larger scale, offering guidelines and tools for constructing solar-powered EV charging station infrastructure.

## Introduction

By 2050, two-thirds of humanity is expected to live in cities^1^ posing a direct threat to urban sustainability and living conditions. As a result, public policies should encourage the construction and maintenance of urban structures that are both land and energy efficient, contribute to environmental care, and improve population health.

Over the last few decades, there has been a substantial increase in the importance of sustainable renewable resources, reduction in consumption of nonrenewable resources, minimization of pollution, and investment in green infrastructure^2^. Accordingly, decarbonization of the electrical sector and widespread electrification of transportation, industry, and buildings are considered to be essential for energy transition. Consequently, investments in renewable energy sources like wind and solar photovoltaics (PV) are growing^3^.

Solar PVs are a well-known source of power among the various renewable energy sources^4,5^. Despite historically limited deployment due to economic and market availability^6^ rapid cost reductions have resulted in an affordable levelized cost for solar power^7–9^. However, since energy production is directly related to the surface area covered, solar farms need a considerable amount of land^10^. Due to a lack of available land, opportunities for large-scale solar PV installations are typically only available as rooftop installations in densely populated areas^11^. However, due to the non-uniform geometry of the buildings, the shade of nearby objects makes it difficult for them to effectively absorb solar radiation. Moreover, contiguous renewables in urban environments can be challenging because of the density and arrangement of the cities’ structures and the ownership considerations of the story buildings^12^. To meet their energy demands, other alternatives should be thus developed to ensure renewables-based energy self-sufficiency of cities^13,14^. It is possible, for example, to repurpose parking lots into solar farms with PV canopies to increase energy production^15^, and preserving the parking spaces’ utility while avoiding the expenses associated with conventional grid growth. This would also allow for long-term energy production while minimizing environmental impact^16,17^.

Near workplaces, shopping centers, parks, recreation and residential areas, open parking lots provide easy access for nearby users. Adding shade structures to existing carports can also improve pedestrian and vehicle safety during extreme weather. Moreover, Du et al.^18^ found that parking spaces offer pre-cooling for vehicles, which can efficiently compensate vehicle power consumption due to less need for air conditioning, particularly at commencement.

Open parking lots were found to have potential technological, environmental, and financial benefits in a study by Nunes et al.^19^ on the energy generating potential of a carport. Malek et al.^20^ proposed a 3kWp carport design with 12 PV modules for charging small urban vehicles with considerable reduction in carbon dioxide (CO_2_) emission. In another study, Merten et al.^21^ investigated the synergy of electric vehicles, PV power plants, and the electricity network in order to enable a substantial market share of electric vehicles while considerably reducing greenhouse gas (GHG) emissions.

Although there are various studies that looked into renewable energy production from different perspectives, the environmental and economic implications of integrating open parking lots with PV-integrated electric vehicle (EV) infrastructure have not been systematically investigated (considering travel distance, neighborhood driving habits, and commonly used EV brands in the market). Furthermore, while other studies mainly focused on a specific site with limited access to certain residents (ex. university campus), this study considers public benefits by connecting the potential visitor flow to the tourist attraction site (knowing that the art center, nearby the study area, is primarily intended for innovative ideas and public interest) and sustainable energy promotion in the proposed solar carport canopy. Open carports, which cover substantial surface areas in cities and are typically underutilized as single-use spaces, present an untapped opportunity to produce energy in urban systems^22^. Furthermore, financial incentives analysis, the synchronic effect of parking fees and carbon pricing, and how these respond to different decision making by drivers, have not been thoroughly investigated, particularly in areas like Taiwan with limited land for solar farms and increasing number of vehicles. This information will be very helpful to legislators as they work to green the transportation industry, which may encounter strong public opposition. The current study provides important strategy and investigation to keep up with the rapidly growing demand for this mode of transportation, especially in light of the recent update to Taiwan’s “Fuel Economy Standards and Regulations on Vehicle Inspection and Administration”, that provides subsidies for users when purchasing EVs^23^.

As electricity demand usually peaks during intense sunlight^24^, Taiwan subtropical climate, with long, hot summers and short, moderate winters, is excellent place for solar panel development. However, despite abundant sun radiation, the country’s population density of 646 persons/km^2^ in 2021 and two-thirds mountainous terrain limit the land area suitable for solar energy installation. Furthermore, roughly 80% of the population lives in urban planned neighborhoods with high development density^25^. Reducing climate impact, enhancing the sustainability of urban ecosystems, and encouraging a low-carbon energy approach should be among the most important urban planning strategies. Solar PV carports paired with EV charging stations can therefore function as an ideal independent source of energy supply that not only helps to reduce GHG emissions, but also benefits suppliers by facilitating market interaction between supply and demand^26^.

This study presents a framework for technical approaches and economic evaluation of carport solar panel shading deployment, as well as feasibility assessment for an EV charging station in Kaohsiung, Taiwan. It methodically addresses resource evaluation, as well as the orientation and setup required for such a deployment. The provided approach and analysis are universally applicable to encourage PV development in similar areas with high PV deployment potential and will benefit city decision makers and academics in this sector. Moreover, the proposed integrated flowchart of the process and elements to consider for the deployment of solar PV carports in future research and decision-making scenarios is a holistic management approach to understand the implications of social acceptance and environmental impact assessment in the development of renewable energy projects. This framework can greatly help to guide future city planning scenarios.

## Results and discussion

### Site description

**Figure 1:**
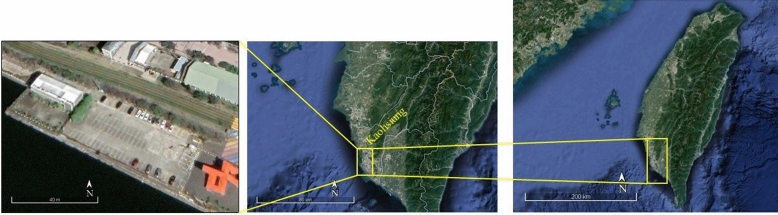
Geographical location of the studied site in Kaohsiung, Taiwan (Google Earth Pro 7.3.3.7786 (2021)).

After a visual evaluation of various car parking lots with the consideration of shadowing impact, a parking lot in Taiwan's Kaohsiung City was chosen where the locations and physical features of selected site are as shown in Fig. 1 and Table 1.

**Table 1:** The studied site details and features.

Geographical location	22°37′10.7"N 120°16′51.9"E
Parking area	1676.56 m^2^
Physical features	•24 h parking lot
	•Car spaces: 84
	•Public paid

### Analysis of solar radiation

**Figure 2:**
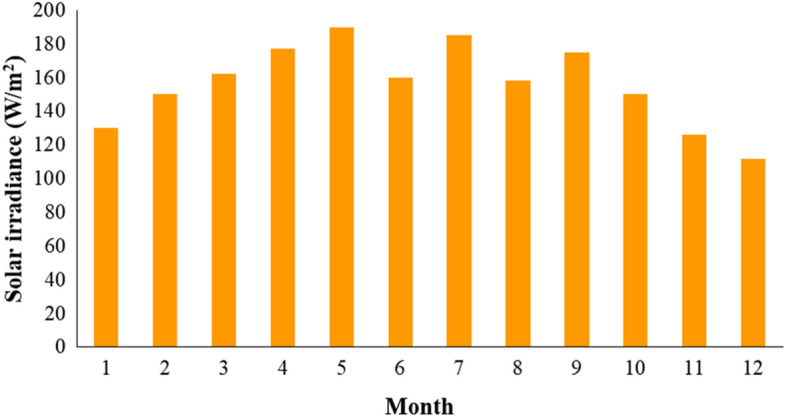
Monthly solar irradiance of Kaohsiung city (adopted from^28^).

Due to abundant sunshine and potential areas that can accommodate solar PV energy installations, solar power is the most applicable renewable energy option for Taiwan^27^. Monthly solar irradiance of Kaohsiung city have been depicted in Fig. 2, confirming southern Taiwan a suitable place for solar panel installation.

Since the development of renewable energy sources is a top priority for Taiwan to ensure a reliable energy supply, sustained economic expansion, and rapid technological progress, the “Five Plus Two” plan was launched by the Taiwanese government in 2016 as part of their efforts to advance renewable energy development^29^. The plan intends for renewable energy to account for 20% of Taiwan’s electricity by 2025, which will lessen the country’s dependency on imported energy sources and a positive effect on the environment. The “Five Plus Two” plan calls for 20 GW of solar power, with 14 GW coming from ground-mounted systems and 6 GW from rooftop systems, providing 66.3% of the total needed energy^29,30^.

### Modeling of the carport canopy

**Figure 3:**
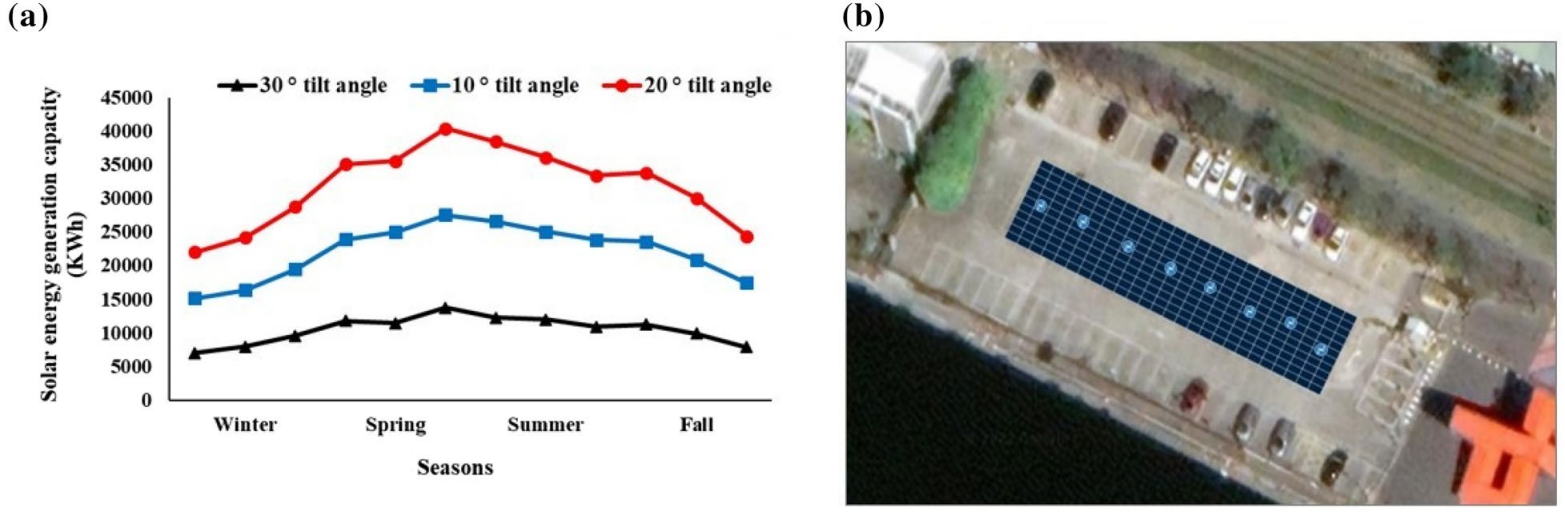
(**a**) Solar PV energy generation capacity in different seasons with various tilt angles; (**b**) Mono-pitch canopy without shading effects at optimum tilt of 20°.

An analysis of monthly PV energy (kWh) production placed at various tilt angles reveals that 20° is the best angle for the examined location, providing the most solar energy generating capacity (Fig. 3a). In addition, the weather and temperature vary the energy production throughout the year, with the summer months being the best for solar energy production since the sun is at its highest and the days are longest. The yearly output of accessible solar energy of the proposed carport canopy is estimated to be 140 MWh by installing 286 solar modules at a 180° azimuth angle facing south (Fig. 3b). The amount of energy produced by solar panels is dependent on factors such as the size, number, sunlight irradiance, and direction of the panels. For example, Badea et al.^31^ designed, dimensioned, and simulated an isolated system for a EVs charging station with PV panels for 45.65 m^2^ generating 5789 kWh/year, with a total CO_2_ emission embedded into 583 kgCO_2_/year. Another study by Alghamdi et al.^22^ showed PV installation with a capacity of ~ 36.4 MWp annually within a university campus in Saudi Arabia with 594,611 m^2^ parking area.

It is noted that since construction of the carport in the current study area is not affected by the surrounding objects and none of the PV modules’ efficiency will be influenced due to the shadow effects, neighboring building and tree shading effects are neglected in this simulation.

### PV-supported charging station for electric vehicles

**Figure 4:**
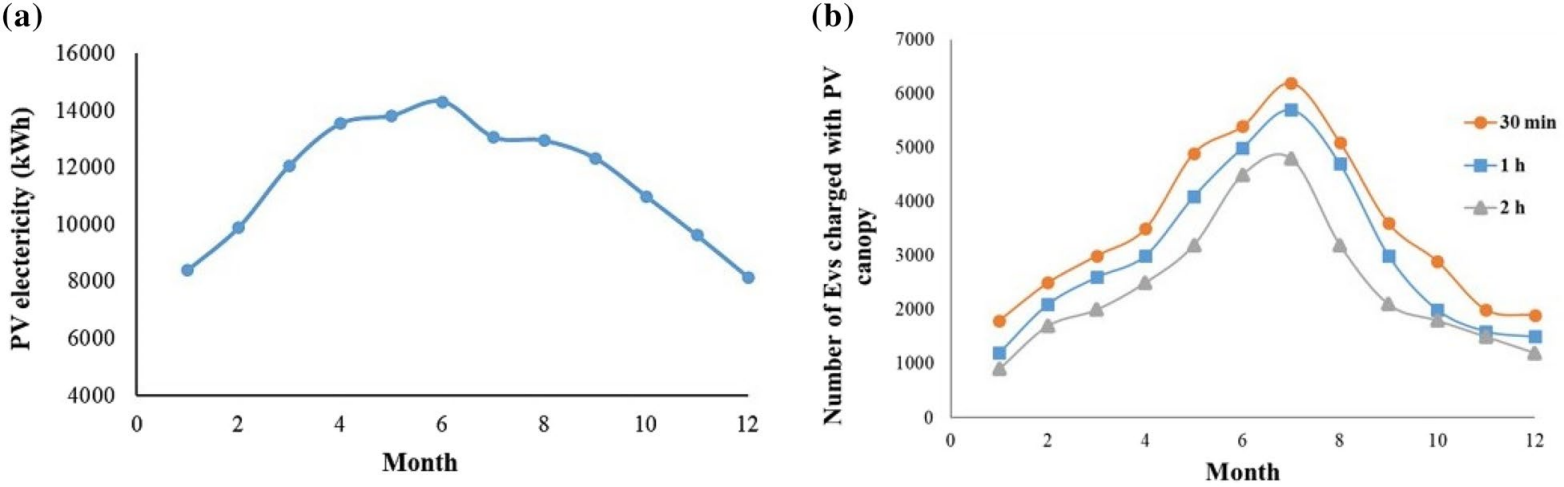
PV electricity (kWh) (**a**) and number of EVs charged with PV electricity (**b**) produced from carport canopy solar power in the study area.

The monthly PV power production (Fig. 4a) is simulated using the geographical and physical characteristics from Tables 2 for the PV canopy area depicted in Fig. 3b.

**Table 2:** PV specifications of the studied site details and the carport canopy features.

Item	Value
PV system size (Kw)	50
PV system lifetime (years)	20
CO_2_ emissions in manufacturing of PV (kg CO_2_ equiv/kWp)	800
Operating temperature (^○^C)	− 40 to + 85
DC System Size^1^ (kW)	5588
Fixed Array Tilt (^○^)	20
Canopy area (m^2^)	525.6
Nominal Capacity of battery (Ah)	189
Battery voltage (V)	12
CO_2_ emissions in manufacturing of battery (kg CO_2_ equiv/kWp)	55
Inverter efficiency (%)	98
Inverter Continuous power (VA)	900
Array Azimuth (^○^)	180
System Losses (%)	14^2^
Number of PV panels	286

^1^As computed by PVWatts after specifying the area.

^2^System losses are calculated by taking into account numerous factors such as soil erosion, rainstorms, light-induced damage, cabling, shade, mismatch, aging, accessibility, dust, and so on.

**Figure 5:**
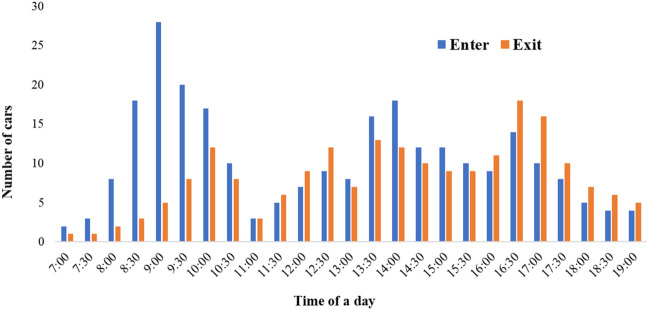
Representive histogram presenting number of car entries and exists per hour during the month March, 2021.

The average solar PV system can generate 1 to 4 kWp, which is sufficient to fully charge a 40 kWh battery electric vehicle in just over eight hours. Nevertheless, the quantity of solar energy available to charge an electric vehicle will vary based on the season and the weather conditions. PV electricity generated from carport canopy solar power (kWh) and the number of EVs charged with PV canopy in the study area have been depicted in Fig. 4. The quantity of charging stations for which the PV canopy can provide 100% of the electricity for 12 h per day of charging, varies depending on the season and and the length of time they spend at the parking lot. Eight charging point with 3.7 kW charging units and minimum of 5 m apart is recommended for the suggested area. Each charger allows simultaneous connection of four cars. To analyze the system's needs, only battery-electric cars with generally larger batteries than plug-in hybrid electric vehicles are examined. The arrival times and lengths of stay of the vehicles evaluated in this study are based on data acquired from a preliminary examination of parking entries and exits in the study region, with averages of 30 min, one hour, and two hours. An example of observational data collected based on the video-recording of the activity of a parking lot during a 12 h period has been shown in Fig. 5.

According to Fig. 4b, the quantity of vehicles that can be charged depends on both the month and the time required to complete the charging procedure. The monthly average quantity of cars that the research region could power with solar energy is 2458, 3017 and 3592 for 2-h, 1-h and 30-min parking time, respectively with a pick number in July for all three scenarios.

Similarly, Deshmuk and Pearce^32^ proposed that the number of cars that can be charged is highly dependent on both the PV canopy surface area and the charging time when investigating the EV charging potential from retail parking lots. In another study in Newark^33^, where the array has a peak power of 300 WP and an inverter efficiency of 90%, the PV integrated in the EV accounted for about 12% of a Chevy Volt’s annual mileage.

### The benefits and motivations of charging EVs with solar power

Table 3 displays the charging capacity and charge time for Taiwan’s most popular electric vehicles. As can be seen, none of the EVs can be fully charged on a standard spending trip. The Tesla model X 100D has the fastest charging time at 6.4 h, while the other two Tesla models take longer. Since slow charging times are discouraging potential EV consumers, it is thus recommended to justify the charging rate strategies for long-term investments, as people evaluate the charging speed of the EV’s onboard charger when planning to use public charging stations regularly. More research is needed to measure this.

**Table 3:** Charging information for EVs.

	Tesla model S 100 dual	Tesla model 3 (long range)	Tesla model X 100D
Battery capacity (kWh)	100	75	100
Battery acceptance rate (kW)	17.20	11.50	17.20
Type of Battery	Lithium ion	Lithium ion	Lithium ion
Time to full charged (h)	8.42	6.50	6.40
Range for full charge (km)	539.13	498.89	416.82
Charge received per charging hour (kWh/h)	9.61	9.61	9.61
Charged kilometers per h	64.18	76.75	65.12

Aside from that, Table 3 displays the quantity of energy gained and the total distance traveled by each of the evaluated EV models after an average charging time of one hour. Within an hour visit, all of the vehicles were able to go at least 60 km on their one-hour charge. This is noteworthy as according to taxi demand analysis, Pier-2 Art Center is among the most demanded tourist attractions^34^ and many Taiwanese residents will take a day trip to the studied park area from neighboring regions, mostly Tainan city which is less than 60 km away from the case study parking lot. According to the study by Chou and Lin^35^ on the inter-city transportation activities using electronic toll collection big data, most cars from Tainan are traveling short-distance and more likely to travel to Kaohsiung downtown area in weekdays and tourist areas in weekends. This suggests that for many EV owners, the drive to the Pier-2 Art Center (the study site) and back could have very minimal automobile-related energy costs. This also emphasizes the benefit that Tesla vehicles presently have and implies a likely performance upgrading for coming EV models, as well as boosting the long-term viability of this technique.

More charging stations should be installed at popular tourist destinations to accommodate the influx of visitors, particularly on the weekends. As distance from activity place to charging station plays a crucial role in EV users’ willingness to charge publicly^36^, the proposed parking lot is an ideal place for installing the solar equipped EV stations. According to Table 3, considering the percentage charge as a function of the amount of time spent, visitors and shopkeepers at the local food street vendors and those who plug in their vehicles while spending time around the art center would greatly benefit from EV charging in a recreation area. Since this art center is one of the most popular tourist destinations, the expansion of green transportation is critical to the development of tourism as well as economic benefits^37^ and will potentially improve urban air quality^38^.

### Emission impact

The battery capacity of the car and the energy sent to it by the charging station determine how many EVs can be charged at a time. From the information on the number of EVs the planned charging station can accommodate, the reduction in emissions was calculated based on how much CO_2_ would have been released if the same quantity of energy had been obtained from the grid (proportional to the contribution of various energy generation sources to the grid). While it is shown that an EV with a grid-powered battery may help clean the environment by reducing GHG emissions^39^, but they considerably increase demand for grid electricity. This extra grid electricity produces GHG emissions because it still primarily derives its energy from fossil fuels. Typically, GHG measurements for EVs only capture emissions savings from the direct combustion of fossil fuels and do not account for indirect emissions linked to the transmission and generation of energy for the grid^40,41^. Alternative energy sources including solar, wind, and biomass are therefore highly commended for their efficiency and reliability^42^. For instance, in Macau, where power is produced by conventional sources, the electric public bus cannot substantially reduce GHG emissions compared with public bus fueled by diesel^43^. Therefore, the benefits of using an EV in terms of GHG emissions are dependent not only on the electric power sources that are utilized to power the EV, but also on its efficiency, range, and modes of operation.

**Figure 6:**
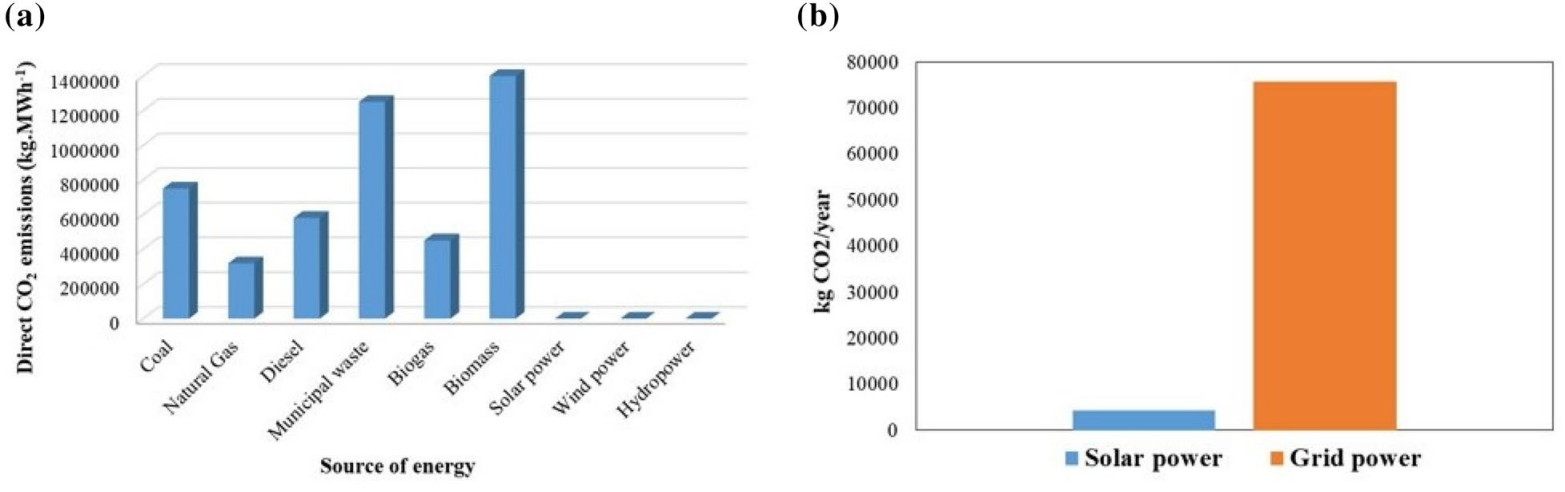
(**a**) Direct CO_2_ emissions (kg.MWh^-1^) related to the source of electricity production, and (**b**) CO_2_ emissions in proposed PV carport canopy and traditional grid system.

Although solar and wind power plants do not release any direct atmospheric CO_2_ during the process of generating electricity (Fig. 6a), the average value of indirect emissions from the system’s individual components based on the specifications of the battery and PV panel characteristics are 26 kgCO_2_ MWh^−1^, 11 kgCO_2_ MWh^−1^, and 29 kgCO_2_ MWh^−1^ for solar, hydro, and wind power generation, respectively^44^. A year of operation would result in a total CO_2_ emission of about 4 ton CO_2_/year from the planned PV system in the current study with applied PV system size, which would generate a yearly output of 140 MWh. As the PV generator does not produce any additional emissions, the emission accounted only for the CO_2_ emissions from the system. The emission factor of grid power is published annually in Taiwan by the Bureau of Energy (BoE), an administrative agency of the Ministry of Economic Affairs (MoEA), and the governing authority of the power system in the country^45^. The emission factor for electricity of year 2020 was 0.502 kgCO_2_ kWh^−1^ in Taiwan^46^. To generate the same amount of power as a PV system, the conventional system would emit about 70 tons of CO_2_, as depicted in Fig. 6b. As a result, solar panels in the current study produced electricity with a CO_2_ footprint that is about 94% lower than that of conventional grid sources of electricity generation. This energy supply design, which incorporates PV panels on an EV charging station, promotes sustainable economic growth by encouraging investment and effectively managing the economy for the benefit of people and the environment^31^.

However, it is important to note that the CO_2_ emission factor for electricity is highly influenced by the power generation mix. To facilitate the transition to a low-carbon energy system, the government of Taiwan has committed to raise the percentage of renewable energy to 20% of total power output by 2025, and the percentage of natural gas to 50%, while reducing the percentage of coal to below 30%. In light of this, it is predicted that traditional grid CO_2_ emissions would drop to around 60 tons per year by 2025, with the emission factor for electricity decreasing to 0.394 kg CO_2_ kWh^−1^. Even though switching to alternative fuels will increase energy use, it has the potential to considerably reduce CO_2_ emissions^47^. Besides, the current emission factor computations are based on the yearly average CO_2_ emission, which represents the carbon intensity of grid-supplied power as a single, static amount throughout the year. However, because the mix of generators delivering energy to the grid is continually changing, the carbon intensity of the system fluctuates throughout the year and during the day^48–51^. Although disregarding this hourly variability may reduce precision^52^, it is unclear from previous research whether this possible bias is considerable or common. Existing research focuses on individual building GHG inventories as case studies, revealing that yearly accounting may skew emission inventories by anywhere from 0.2 to 35% when compared to hourly accounting^50,53–55^. Since the only official available carbon emission data for Taiwan’s power generation is annual based^46^, and consequently hour-based inventory data was not accessible, an error range was applied for possible over- or under-estimate of annual-average carbon emission with highest possible error based on literatures, demonstrating the range of CO_2_ grid emissions to be between 45.5 and 94.5 tons of CO_2_ to generate the same amount of power as a PV system based on emission factors report for electricity of year 2020. Also, GHG emissions is calculated as the sum of carbon emission from diesel fuel/gasoline, and electricity consumption. Therefore, unlike solar panels in this study, emissions from construction of power generating utilities are not included in the grid emission factor calculations. On the other hand, while solar panels do not generate GHG emissions when in operation, when considering their life cycles, emissions are predominantly identified in the manufacturing process of component parts as direct emissions originating from the component manufacturing process or indirect emissions from the consumed energy associated to the solar panels production country’s electricity generation emission factor. Since information on energy consumption for each manufacturing process depends on the manufacturers, and the emission factor information depends on the voluntary information of the country^56–58^, subjected to some degree of uncertainty to the available datasets, life cycle consideration of solar panels is not considered in the emission calculation of the present study. Further research on total emission generated by the solar PVs during the life cycle is recommended since it enables society to be aware of the environmental impacts associated with the product during its life cycle.

### Financial analysis for vehicle owner

The EV charging financial analysis provides customers with important information about the financial success of electric vehicle charging installations. The average cost of home charging for Taiwanese citizens is about 105 US$/kWh per year, assuming that every car is charged at home during nighttime without the usage of solar energy^59^. The vehicle owner will therefore benefit financially from any yearly parking rate less than 105 US$ for a PV-based charging infrastructure. Nonetheless, this annual fee does not include the construction of a charging station and communication infrastructure at home. Given that the average hourly price for public parking lots in Taiwan is roughly 0.7 US$, employing a PV carport canopy can greatly contribute to the financial benefits of Taiwanese residents who live nearby or frequently commute to the study area.

**Figure 7:**
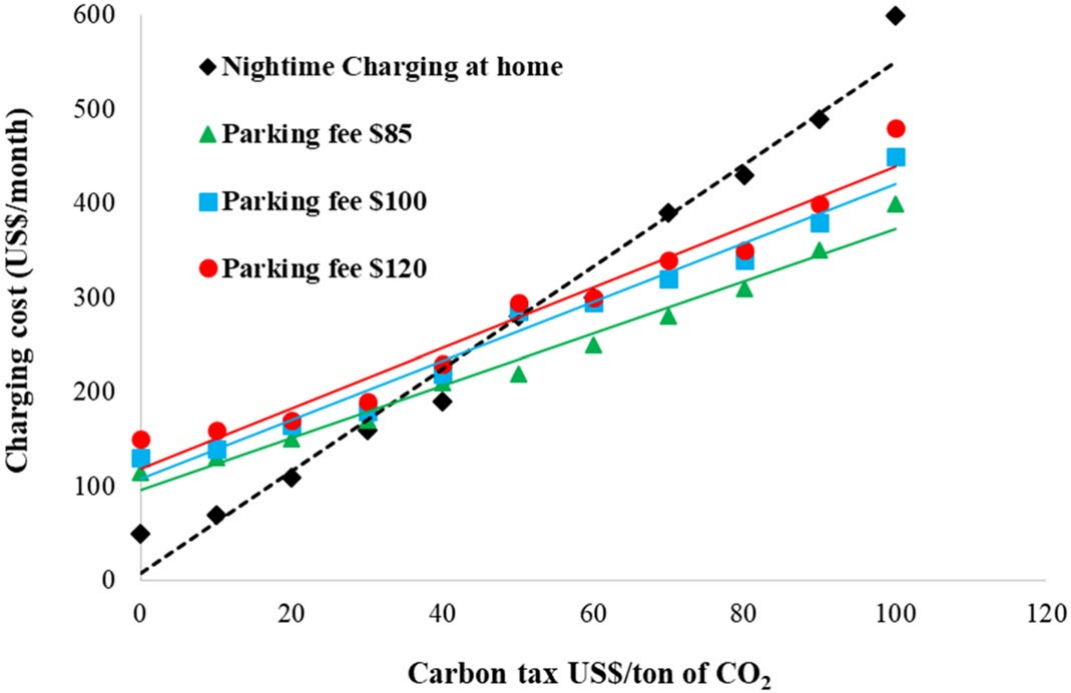
The effect of various carbon tax rates and parking fees on EV charging costs.

It is essential to consider the benefits of clean energy sources by employing PV-powered charging. Policies encouraging clean energy suggest that a carbon tax may be widely implemented in the future. The carbon tax is in effect in a few countries, and Taiwan's EPA has suggested a low carbon tax for large polluters to be imposed in 2023 to counter the EU’s carbon border adjustment mechanism (CBAM) and push domestic emissions to zero by 2050^60^. With the intention of preserving the environment and boosting tax income, a carbon tax makes non-carbon technologies more competitive with respect to conventional fossil fuel burning^61^. Although a metric ton of CO_2_ was priced at US$10 following a study of major carbon emitters in Taiwan on carbon pricing^62^, an international expert commission recommended a global carbon price of US$40–80/tCO_2_ by 2020 and US$50–100/tCO_2_ by 2030^63^. Figure 7 depicts the effects of changing carbon tax rates and parking fees on EV charging costs. The solid lines depict the cost of charging per vehicle for different parking fees utilizing PV-based charge in relation to the carbon tax rate. The dashed line represents the monthly cost of charging an EV overnight at home. Solid and dashed lines intersect at the breakeven point, where the difference between solid and dashed lines represents the vehicle owner’s profit or loss at the particular carbon tax rate and parking fee.

For example, with a $50 carbon tax and $85 monthly parking fee, an EV owner can save around $60 by adopting PV-based EV charging. This can highlight the need of boosting Taiwan’s carbon price, which can help clean energy companies compete with higher-carbon industries. By putting a price on carbon, emitters are forced to face the environmental consequences of their activities and are driven to reduce their carbon footprint^64^. However, making carbon taxes more acceptable to the public is critical since it is an efficient way of motivating GHG emission reductions. Although the energy transition is a critical part of the policy response to climate change, little is known about the factors that influence public opinion about it^65^. Greening the transportation sector may face substantial resistance from the public. Since the general public lacks access to the empirical instruments of policy evaluation, communication strategies must be adjusted accordingly^66^. Further socioeconomic studies should be conducted in the future to examine public acceptance and practical remedies in the suggested taxation plans.

### Integrated framework

**Figure 8:**
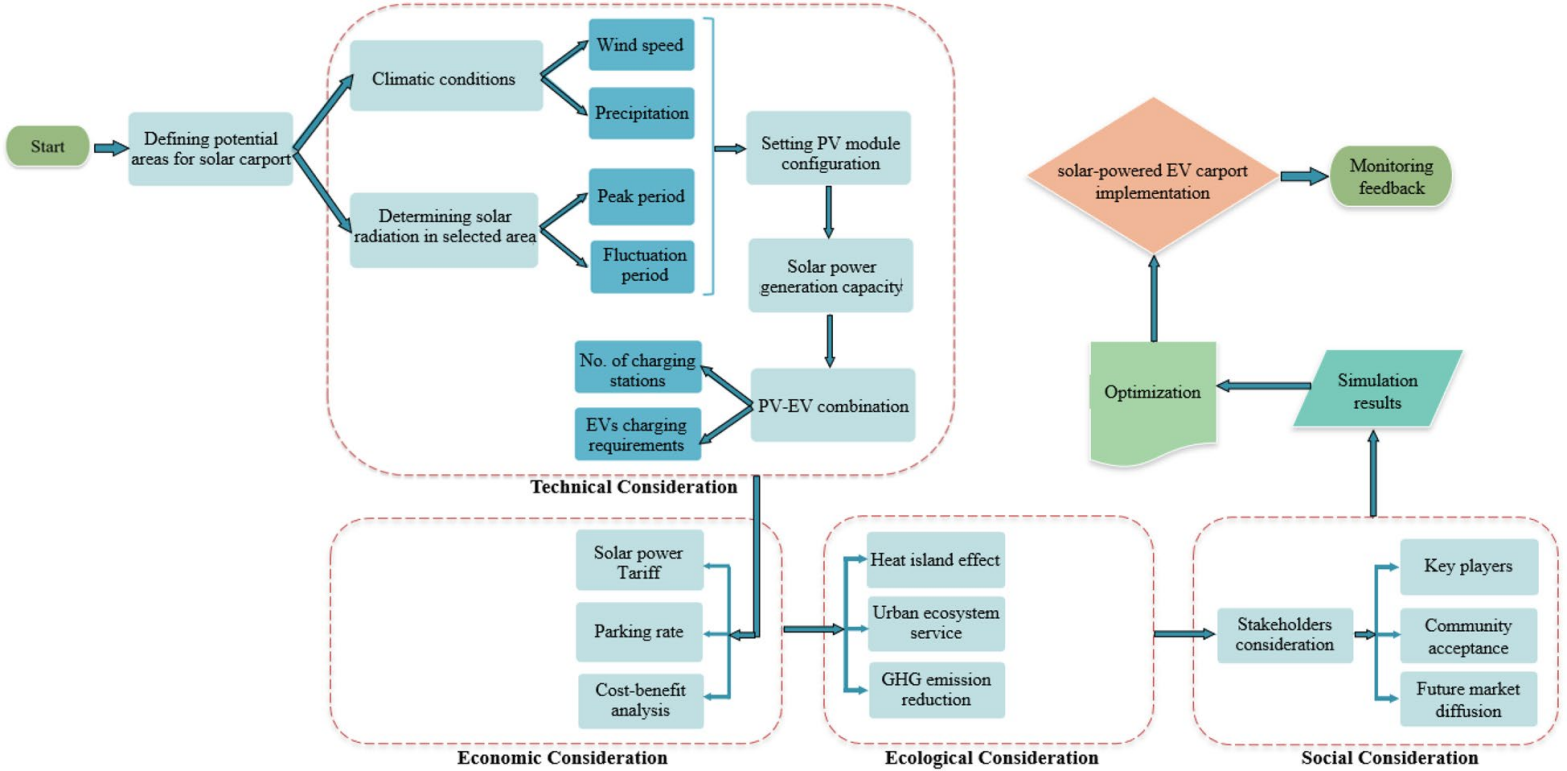
Flowchart illustrating the configuration of solar power carport with EV charging potential.

Multiple social and economic aspects must be considered when deploying a solar-powered carport with EV charging capability. This study developed an integrated flowchart of the process and elements to be addressed for the deployment of solar PV carports in future research and decision making scenarios (Fig. 8). While the present study focuses primarily on the technical and economic analyses, the suggested integrated framework allows for additional research on the net consequences, taking into account potential environmental and social factors. Despite the efficiency of the technology and the rising number of case studies demonstrating its viability, insufficient policy support has slowed the renewable energy industry^66^. In fact, public support is crucial for a successful renewable energy policy implementation^67^. Therefore understanding the implications of social acceptance and environmental impact assessment enables us to develop strategies that encourage the development of renewable energy projects by minimizing public opposition^68^. As seen in Fig. 8, the layout of a solar power carport must take into account a number of technological, economic, ecological, and social aspects. To effectively justify renewable energy projects in city planning scenarios, more research is needed to analyze the interaction between socio-environmental elements (such as stakeholders' considerations and urban ecosystem service) and techno-economic analysis.

### Potential drawbacks

Although the proposed solar carport canopy demonstrated adequate efficiency in producing the electricity needed to power the EVs in the proposed parking lot, in practice, there is always some degree of uncertainty related to future EV charging scenarios. This uncertainty may apply to the timing of the EV arrival or the intensity of the energy demand^69^. Predicting the demand for EV charging stations in the absence of actual usage data presents a challenging modeling problem. Assumptions about vehicle numbers and types, arrival times, average distances, and charging times are needed to create a load profile for the charging station. The proposed model in this research relied on the exact prediction of future solar production and EVs demand over a 12 h horizon in the study area. However, this is not practically feasible scenario because none of these can be determined with certainty in advance. Still, this method can serve as a benchmark for the proposed system's efficiency, regardless of how precise the forecasts may be. However, measures that can effectively address uncertainty across a variety of situations can lower down the model's error and uncertainty and increase energy production efficiency. Several data-driven and analytical approaches to assessing uncertainty have been published. For instance, Zhang et al.^70^ analyzed the charging behaviors and charging times of various groups in order to create a charging load model by means of Monte Carlo simulation. Islam et al.^71^ modeled the daily load profile for EVs in an office setting using a stochastic technique. Ghotge et al.^69^ developed a smart charging method based on Model Predictive Control (MPC) that accounts for this variability in the arrival time of EVs and the total energy demand. Since these methods are beyond the scope of this study and will need further research, the potential shortcomings of the proposed solar carport canopy with EV charging potential are outlined in this section.

The risk of insufficient charging is the most important. Uncertainty is introduced by solar PV’s intermittent nature in this context, and the research area’s dramatic seasonal variation—winter irradiance is around 1.5 times lower than that of summer months, for example (Fig. 2)—is predicted to be the primary source of insufficient charging sessions. Even with a sizable battery energy storage system, overcoming this challenge in the absence of grid connectivity is challenging. The need to reduce energy use during the warmer months is another drawback of this design. The system is scaled according to the amount of available PV installation area per parking spot. In July, the best performing month in terms of electricity production in the study area, the average solar PV system can generate up to 4 kWp, which is sufficient to fully charge a 40 kWh battery electric vehicle in over eight hours. This will cause a high proportion of EVs sitting unused in the parking lot and a large amount of energy being wasted. This production cut represents a clear underutilization of existing capacity and should be avoided if feasible, indicating the necessity for an alternative solution in favor of reducing curtailment.

## Conclusion

The study looked into the viability of employing solar PVs to meet the energy needs of an EV charging station in a public carport in Kaohsiung, Taiwan. The technique established is worldwide relevant to promote configuring PV arrays and selecting sites for PV development, with carport-mounted PVs contributing to national renewables and low-carbon goals. The results demonstrated the feasibility and sustainability of solar canopies for parking spaces in similar areas, indicating that large open car parks in urban areas present a substantial opportunity for the use of renewable energy in this context. The research also looked at the efficiency of charging the most popular models of Tesla's EVs on the Taiwanese market. After spending an hour charging in the solar-covered parking area equipped with EV chargers, all of the examined car manufacturers were able to go at least 60 km. Furthermore, the PV system’s CO_2_ emissions were 94% lower than those from standard grid energy sources, which greatly contributes to reducing environmental impact through effective use of land resources. Because the study area is one of the most popular tourist destinations in southern Taiwan, financial benefits for automobile owners were also researched to determine the influence of various carbon tax proposals and parking fees on vehicle owner profits. The analysis suggests that PV-powered carports would potentially benefit vehicle owners more than home charging if carbon pricing would be applied. Due to their capacity to replace CO_2_ emissions from the electrical grid, the establishment of a carbon tax make these renewable charging stations more appealing and advantageous.

## Methodology

### Analysis

For this work, Helioscope and PVWatts software are employed as a simulation and design program. Folsom Lab USA introduced HelioScope^72^ for developing solar systems, enabling designers to do a comprehensive design with a single package. HelioScope requires the location’s address, array setup, PV module information, and inverter specification as its primary inputs. Additionally, it provides a tool for calculating the shadows created by buildings, trees, and towers. PVWatts, on the other hand, is one of the well-designed web-based software tools used for calculating the energy production from a PV system at any location based on typical meteorological data for the selected site^73^. The PV cell temperature is calculated for every hour of the year by the PVWatts calculator. Hourly DC energy output is determined by feeding in data about the DC rating of the PV system, the amount of incident solar radiation, and the temperature of the PV cells.

There are two general kinds of carport canopies: single row canopies and double row canopies. The most frequent style, the double row carport, is proposed in this study since it requires less area for installation, has a low cost of cabling, and also smaller iron frame.

**Figure 9:**
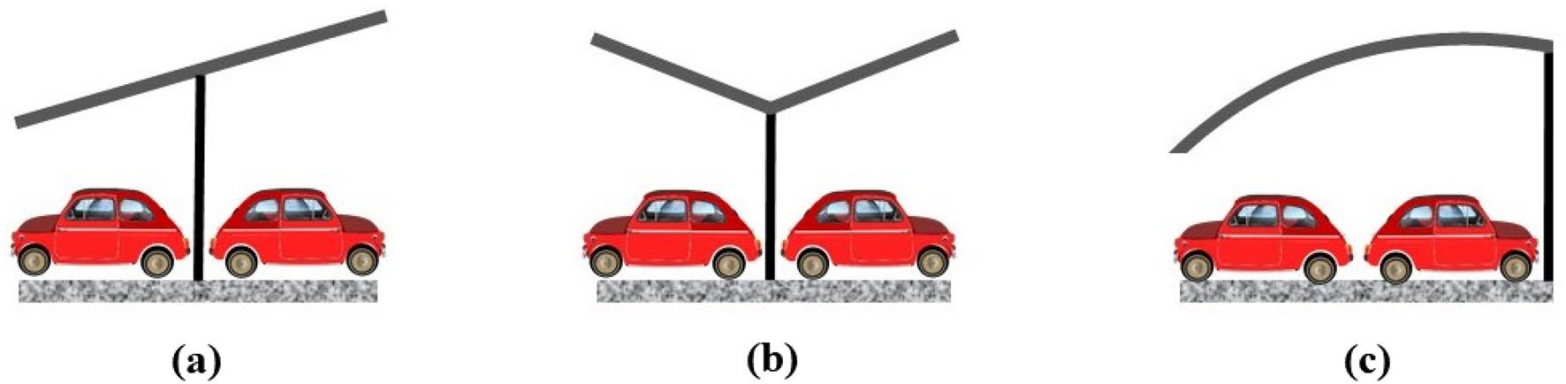
Design of various carport canopies as (**a**) Mono-pitch canopy, (**b**) Duo-pitch canopy, and (**c**) Barrel arch canopy.

A mono-, duo-pitch, or barrel arch canopy is recommended to optimize solar output from the area to be covered based on the available space, orientation, and possibility for shading of the parking lot (Fig. 9).

The optimum method for maximizing a PV array’s energy output is to tilt it at the ideal tilt angle^74,75^, and PV panels produce the most energy when they are installed or situated facing away from the sun. This study examined tilt angles to maximize PV energy output. The study considers systems tilted at 10°, 20° and 30°.

### Climatic conditions

Tropical weather prevails in Taiwan’s south, where annual average temperatures range around 24 °C^76^. One of the most abundant natural resources in the country is sunlight due to its location in a subtropical area surrounded by oceans. As a result of the Tropic of Cancer passing through Central Taiwan, there are long daylight hours and little angle of sunlight deflection^77^. According to estimates, Taiwan receives 1.7 × 10^11^ kW/h of solar energy every day^78^.

### Energy generation of solar PV systems

Weather conditions^79^, local geographical features^80^, type of solar PV used^81^, and site conditions (shading effects of nearby objects and radiation reception)^80^ all have an impact on the energy generation of solar PV systems. The amount of solar energy potential, is derived from HelioScope simulations based on the potential of sunlight resources of the area and PV characteristics. After determining the number of modules, inverters, and installed power potential, simulations is conducted to evaluate the energy production generated from the parking lot using the following equation^82^:

1 $$\begin{aligned} E_{grid} \; & = E_{A} \eta_{inv} \\ & = E_{P} \left( {1 - {\uplambda }_{P} } \right)\left( {1 - {\uplambda }_{c} } \right)\eta_{inv} \\ & = S\eta_{P} \overline{H}\left( {1 - {\uplambda }_{P} } \right)\left( {1 - {\uplambda }_{c} } \right)\eta_{inv} , \\ \end{aligned}$$

where ${E}_{grid}$ is the net energy transferred to grid (kWh); ${E}_{A}$ depicts the PV array energy generation (kWh); ${E}_{P}$ represents the PV array delivering energy (kWh); ${\eta }_{inv}$ is the inverter efficiency (%);${\uplambda }_{P}$ is miscellaneous PV array losses (%),${\uplambda }_{c}$ refers to the conditioning losses for PV array (%);$S$ is the area of array (m^2^); $\overline{H }$ is the average hourly irradiance on the PV array panel (kWh); and ${\eta }_{P}$ is the array efficiency (%).

The number of charging stations, S, which can be installed at the selected parking lot is then given by:

2 $$S=\frac{{E}_{d}}{r\times {h}_{d}},$$

where ${E}_{d}$ is the PV-generated electricity per day measured in kWh by the PV canopy, $r$ is the electricity transferred per hour by the charged station (kWh) and ${h}_{d}$ is the estimated number of hours a given parking lot space is used per day. Here ${h}_{d}$ is estimated to be 12.

The number of kilometers (*K*) that an EV owner could be expected to travel based on a charge receiving at the carport canopy is determined by:

3 $$K=\frac{{r}_{max}\times R}{C}$$

where ${r}_{max}$ is the maximum charge rate (kWh), which can be set by the limitations of either the EV or the charging station, $R$ is the EV range in km and $C$ is battery capacity in kWh.

### Technical considerations for Solar EV charging station

**Figure 10:**
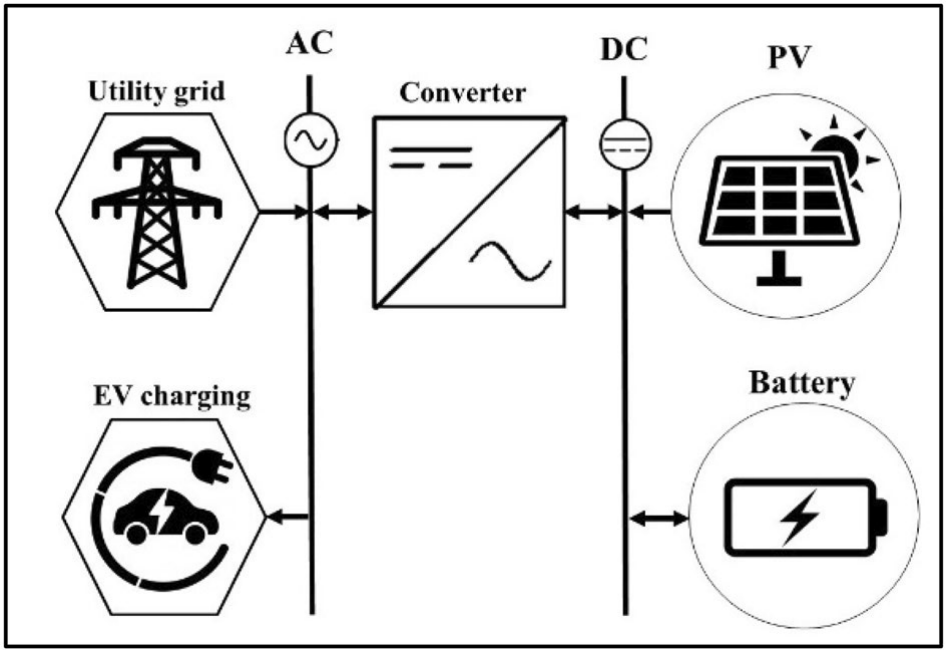
Schematic of a solar-powered EV charging station linked to the grid.

As shown in Fig. 10, the general design of an EV charging station powered by PV consists primarily of the PV system, the DC-AC and AC-DC bi-directional converter, batteries, the utility power grid, and EVs. This arrangement is the ideal design for the EV power supply model to lower the cost of electricity, solar power systems, batteries, and carbon emissions^83^. In daytime, the EV may charge straight from the solar power system, and at night or in extreme weather situations, it can charge from the utility grid. When there is no solar or grid power, batteries in the electric vehicle charging station are intended to satisfy minimal energy storage and backup requirements, which lowers the overall system investment cost^31^.

The concept of a solar carport is to cover parking spaces with PV canopies to meet onsite energy needs. Wherever a parking lot is required or already exists, this solution can be installed. The parking lot in the current study is already located in the Kaohsiung Port basin (Pier-2 Art Center) and nearby a unique public plaza where artists and the general public can exchange ideas. It is currently regarded as one of Kaohsiung’s most important cultural attractions and a popular tourist destination that contributes to the city’s economic growth^84^. Due to the increasing number of vehicles and tourism-related activities in the region, there has been an increase in the number of vehicles looking for parking, especially for short stops. The plan of Kaohsiung City government to increase the number of EV charging stations in public parking lots by 5% per year^85^ can further support the selection of the study site and the proposed approach for the solar carport canopy.

In general, one parking space is estimated to be 15–18 m^2^^19,22^. In this study, a double-row size of eighteen carports is proposed for the examined location. The length of the double row carport is 15 m and the width is 7 m to accommodate six cars per row. The entire space of 525.6 m^2^ is available at the carport shade for the generation of maximum power and efficient use of carport canopies.

The energy consumed by EV charging stations will be compared to the electricity produced by PV canopies using available solar flux to estimate the number of EVs that can be charged based on the average time a car is parked in the studied parking lot, charging rates, and charging capacity of the EV’s.

## Data Availability

The datasets generated and/or analyzed during the current study are available from the corresponding author on reasonable request.
